# Skull Impact on Photoacoustic Imaging of Multi-Layered Brain Tissues with Embedded Blood Vessel Under Different Optical Source Types: Modeling and Simulation

**DOI:** 10.3390/bioengineering12010040

**Published:** 2025-01-07

**Authors:** Xi Yang, Chengpeng Chai, Yun-Hsuan Chen, Mohamad Sawan

**Affiliations:** 1 CenBRAIN Neurotech Center of Excellence, School of Engineering, Westlake University, Hangzhou 310030, China; yangxi@westlake.edu.cn (X.Y.); chaichengpeng@westlake.edu.cn (C.C.); 2 Institute of Advanced Technology, Westlake Institute for Advanced Study, Hangzhou 310024, China

**Keywords:** brain imaging, optical simulation, Monte Carlo simulation, acoustic simulation, k-wave, skull, blood vessel

## Abstract

Skulls with high optical scattering and acoustic attenuation are a great challenge for photoacoustic imaging for human beings. To explore and improve photoacoustic generation and propagation, we conducted the photoacoustic simulation and image reconstruction of the multi-layer brain model with an embedded blood vessel under different optical source types. Based on the optical simulation results under different types of optical sources, we explored the characteristics of reconstructed images obtained from acoustic simulations with and without skull conditions. Specifically, we focused on the detection of blood vessels and evaluated the image reconstruction features, morphological characteristics, and intensity of variations in the target vessels using optical and acoustic simulations. The results showed that under the initial PA signals, the types of optical source types corresponding to the strongest and weakest photoacoustic signals at different positions within the target region were consistent, while the optical source types were different in the reconstructed images. This study revealed the characteristics of acoustic signal transmission with and without skull conditions and its impact on image reconstruction. It further provides a theoretical basis for the selection of optical sources.

## 1. Introduction

With the developing imaging technologies in neuroscience studies, human brain imaging is becoming an important research field to discover the secrets of the brain [1]. With the advantages of optical specificity and acoustic imaging depth, photoacoustic imaging (PAI) as a developing technique has achieved multi-scale brain imaging [2]. Although photoacoustic imaging has achieved functional imaging in small animals with the skull, functional photoacoustic imaging in humans is still not being conducted on the skull [3]. The skull, with high optical scattering and acoustic attenuation, is a great challenge for PAI in human beings [4].

Due to optical propagation being the first step for PAI, many researchers have worked on analyzing the skull’s influence and improving optical propagation [5,6,7]. To improve optical propagation, we also conducted research on the multi-layer brain model under different optical source types using the Monte Carlo method for optical simulation [8]. However, research on optical transmission alone is not enough for the exploration of the PAI in the brain. There are still requirements for understanding acoustic propagation and image reconstruction after photoacoustic generation to solve the problem of the acoustic aspect.

Addressing the technical challenge of strong acoustic attenuation and distortion caused by the human skull (~8 mm), Liang et al. conducted a numerical analysis to study photoacoustic transmission through the layered human skull (forward model) and image formation (inverse problem) [9]. Kneipp et al. in Razansky’s group were the first to investigate the various acoustic distortions caused by mouse skulls and their impact on photoacoustic microscopy (PAM) images [10]. Also, Estrada et al. employed photoacoustic point sources to estimate the insertion loss of the skull and investigated the acoustic wave propagation through human and mouse skulls [11,12,13]. In other groups, Mohamadi et al. conducted a theoretical analysis of the acoustic attenuation and dispersion caused by the skull in 2017 [14]. In recent years, deep learning methods like U-net [15] and improved U-net [16] were researched to improve the image quality caused by acoustic influences. Therefore, we would like to further develop PAI research to consider acoustic simulation and image reconstruction when exploring the influence of the optical source types.

In this paper, we conducted the photoacoustic simulation and image reconstruction of the multi-layer brain models with an embedded blood vessel under different optical source types. The models with blood vessels aimed to better understand the object imaging inside the brain. First, the optical simulation was performed with the illumination of sixteen optical source types based on the Monte Carlo method. Then, photoacoustic propagation was simulated with or without the skull conditions. After that, image reconstruction was conducted, and the results were analyzed based on the characteristics of the reconstructed images.

## 2. Materials and Methods

### 2.1. Brain Model

**Figure 1:**
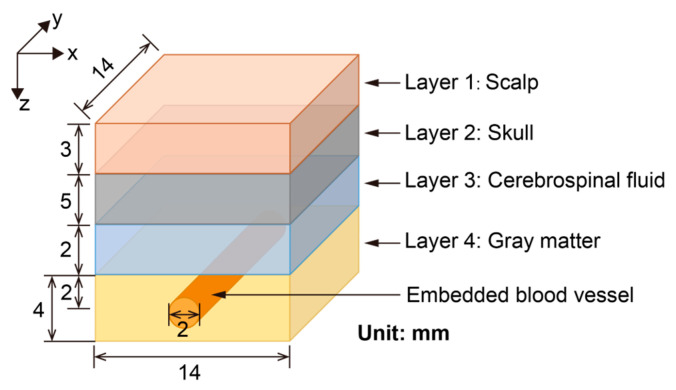
Structure of a simplified brain model with four layers and an embedded blood vessel.

The brain is a complex organ that has different tissues inside the head. Complex brain models can lead to the consumption of computation and time. Thus, we defined a simplified four-layer brain model for optical simulation and acoustic simulation, which meets the requirements of our research. The four layers of the brain model include the scalp, skull, cerebrospinal fluid (CSF), and gray matter. As shown in Figure 1, the thicknesses of the four brain tissues, scalp, skull, CSF, and gray matter, were 3 mm, 5 mm, 2 mm, and 4 mm, respectively [17]. Moreover, considering the diameters of the aorta (25 mm) and capillary (8 μm) [18], a blood vessel with a diameter of 2 mm was embedded in the center of the gray matter layer. A 14 × 14 × 14 mm^3^ volume was set to build a simplified but representative brain model. The volume of the model contained 140 × 140 × 140 grids and the size of each grid was set at 0.1 mm. The optical properties of each brain tissue can be seen in Table 1 [19].

### 2.2. Various Types of Optical Sources

**Figure 2:**
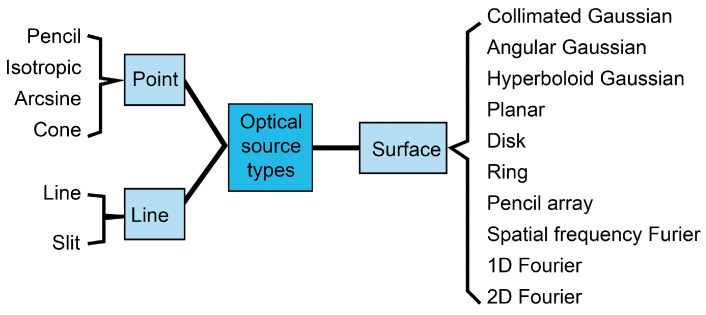
Optical source types into three categories of point, line and surface.

In this research, we explored the influence of the optical source types on photoacoustic (PA) images. Sixteen optical source types are used for optical simulation research (Figure 2). Different types of optical sources include pencil, isotropic, arcsine, cone, slit, line, collimated Gaussian, angular Gaussian, hyperboloid Gaussian, planar, disk, ring, pencil array, spatial frequency Fourier, 1D Fourier, and 2D Fourier sources. The optical sources were set as *x* = 7 mm, *y* = 7 mm, and *z* = 0 mm. The propagation direction was set in the Z direction. The length, thickness, diameter, and distance of those optical source types were consistent as 1 mm. The angle was set as π/6. Other parameters of the optical source types can be seen in the table of the reference [8].

### 2.3. Optical Simulation

Initially, the optical simulation was conducted using the open-source MATLAB toolbox named ‘mcxlab’ [20], which is designed based on the Monte Carlo method in 3D space. The optical fluence was simulated from the optical simulation. Then, the optical absorption was calculated based on Equation (1) [21]:

(1) $$A=\mu_{a}F.$$

where *A* depicts the optical absorption, *F* represents the optical fluence, and *μ_a_* represents the absorption coefficient.

### 2.4. Acoustic Simulation

In this research, the acoustic simulation was conducted based on the optical simulation in the plane of *x* = 7 mm and *y* = 7 mm, which was set as the initial PA signal. The initial pressure for the simulation was calculated based on the optical absorption derived from the optical simulations. The simulation work was finished using the MATLAB k-wave toolbox based on the k-space pseudo-spectral method [22]. According to the optical simulation, the acoustic simulation volume contained 140 × 140 grids in 2D space. The size of each grid was set at 0.1 mm. A line ultrasound transducer array was used in the acoustic simulation, which consisted of 140 elements of the same size as the model grid. The center frequency of the transducer was 5 MHz, and the bandwidth was 100%. A total of 4096 samples were computed at each transducer location with a temporal resolution of 12.5 ns.

In the acoustic simulation, we considered the influence of the acoustic property of the skull. So, we made two acoustic simulation models: one for acoustic homogeneity and the other for acoustic heterogeneity. In the acoustic–homogeneous model, the acoustic property of the soft tissue was similar to the acoustic property of the water (sound speed: 1500 m/s; density: 1000 kg/m^3^) [23]. To account for the acoustic–heterogeneous model, we included the skull region (sound speed: 2190 m/s; density: 1800 kg/m^3^) [15] and the other regions using the parameters for water (sound speed: 1500 m/s; density: 1000 kg/m^3^).

### 2.5. Image Reconstruction

Delay and sum (DAS) is a beamforming algorithm commonly used for PA image reconstruction [24]. The linear ultrasound transducer array with *N* elements was used to receive PA signals. The received PA signals of each component were denoted as *p*(*t*). The reconstructed PA signal *I*(*x*, *z*) at the position (*x*, *z*) can be thought of as the summation of PA signals from all elements. According to the speed of sound and the distance between the object’s position to the ultrasound element *i*, the propagating time Δ*t_i_* can be calculated. Then, based on the sampling rate of the ultrasound array, the PA signals from all elements can be extracted and added to the reconstructed PA signal *I*(*x*, *z*) at a specific position (*x, z*), which is depicted by Equation (2):

(2) $$I(x,z)=\sum_{i=1}^{N}p_{i}\left(\Delta t_{i}\right)$$

During the image reconstruction, the grid size was a 140 × 140 grid with a 0.1 mm pitch. The element number of the ultrasound array was set at 140. A total of 4096 samples were computed at each transducer location with a temporal resolution of 12.5 ns. In the acoustic–homogeneous model, the speed of sound was defined as water of 1500 m/s. In the acoustic–heterogeneous model, the region of the skull layer was defined differently with the speed of sound of 2190 m/s, which is the same as the setting in the acoustic simulation.

### 2.6. Image Evaluation

To evaluate the results of the images, we analyzed the initial PA signal from the results of the optical simulation, sensor data from the acoustic simulation, and reconstructed PA signal from the image reconstruction with two different models. Firstly, the 2D images were analyzed under the sixteen different optical source types. The initial PA images, sensor data, and reconstructed images were analyzed. Secondly, the results in the Z and X directions were analyzed under a specific optical source type (as an example of a pencil source). Meanwhile, the models considering or without considering the acoustic properties of the skull were compared. Thirdly, the initial PA signals, the reconstructed PA signals without the skull, and the reconstructed PA signals with the skull under the illumination of the sixteen optical source types were analyzed. The maximum and minimum values of the results and corresponding optical source types were extracted.

### 2.7. Overview of Method

**Figure 3:**
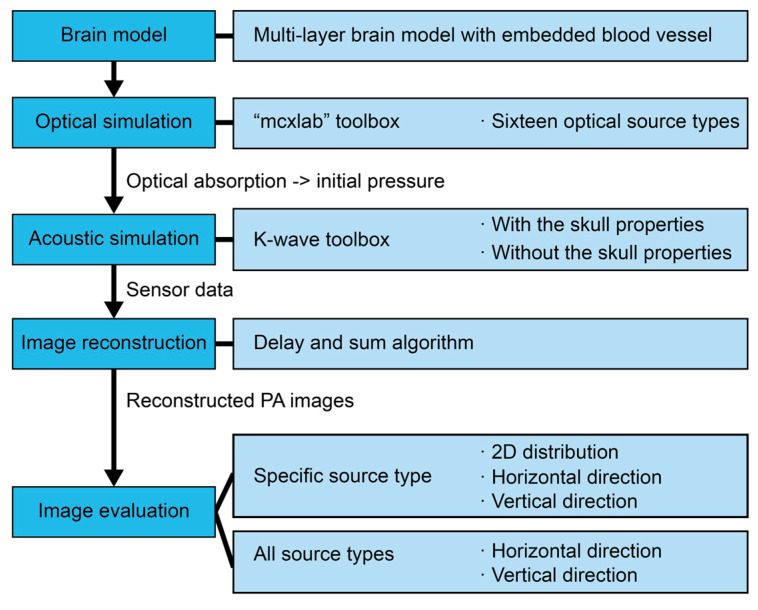
Schematic of research flow in this study.

Figure 3 offers an overview of the research process. In this research, we built a multi-layer brain model with an embedded blood vessel to analyze energy distribution and object situation. Based on the brain model, sixteen optical source types were individually set for the optical simulation using the Monte Carlo method. Next, according to the optical absorption, the initial pressure was set for the acoustic simulation using the k-wave toolbox to acquire the PA signal from the ultrasound sensor. During the acoustic simulation, two conditions with or without the skull properties were considered for simulation. Then, the sensor data were reconstructed into PA images using the DAS algorithm. Finally, the PA images were analyzed in the PA signal distribution in 2D space, horizontal, and vertical directions under the specific illumination type as the example of a pencil beam. Meanwhile, all PA signals of sixteen optical source types were also compared in two directions, and the leading sources were summarized.

## 3. Results and Discussion

### 3.1. Distribution of 2D Images

**Figure 4:**
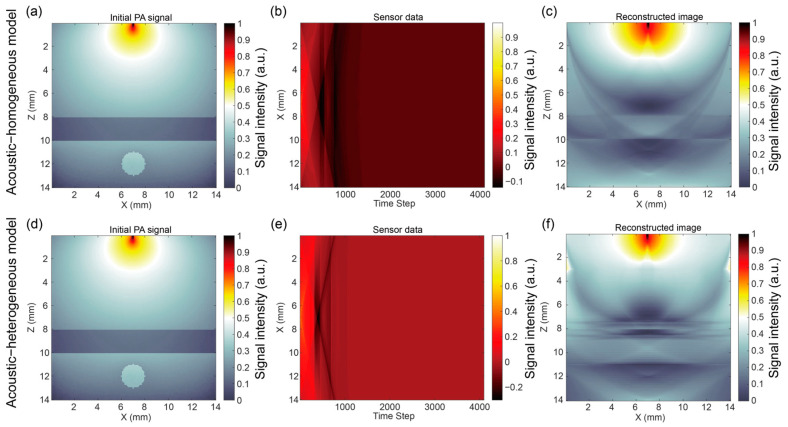
Simulation and reconstruction results of multi-layer brain tissues embedded with blood vessels under the illumination of a pencil beam. Homogeneity of acoustic properties without considering the skull in (**a**–**c**): (**a**) initial PA signal from optical simulation, (**b**) sensor data from acoustic simulation, and (**c**) reconstructed image; heterogeneity of acoustic properties considering of skull (**d**–**f**): (**d**) initial PA signal from optical simulation, (**e**) sensor data from acoustic simulation, and (**f**) reconstructed image.

In this part, the results of the pencil beam (Figure 4), as an example, were analyzed considering the initial PA signal from the optical simulation, the sensor data from the acoustic simulation, and the reconstructed image by the DAS algorithm. Meanwhile, the acoustic simulation was concerned with or without the acoustic properties of the skull. The simulation without the acoustic properties of the skull was defined as the acoustic–homogeneous model while the simulation with the acoustic properties of the skull was defined as the acoustic–heterogeneous model.

Figure 4 illustrates the simulation and reconstruction results of multi-layer brain tissues embedded with blood vessels under the illumination of a pencil beam. Based on the initial PA signal from the optical simulation results, it is found that the incident light spots in the reconstructed image Figure 4c,f were relatively diffused than the initial PA signal at the surface region. The signal in the CSF layer was weak and obvious between the skull and gray matter layers. The object of the blood vessel was more obvious than the gray matter, which had a stronger PA signal.

Meanwhile, we compared the results of the acoustic–homogeneous and acoustic–heterogeneous models without or with the acoustic properties of the skull. It can be found that the object of the blood vessel in Figure 4c without considering the acoustic properties of the skull was nearly circular with a strong signal at the boundary of the top and bottom. The reconstructed image of Figure 4c had a similar layer as the input initial PA signal, but had artifacts in the ellipse shape, which may be due to the limited signal received by the linear ultrasound sensor.

Moreover, in the acoustic–homogeneous model, the characteristics of the layers of CSF to gray matter were calibrated after considering the speed of sound of the skull layer in Figure 4f, which had characteristics that were not like the reconstruction without calibration being moved up. However, there are more artifacts around the CSF layer in the reconstructed image of the acoustic–heterogeneous model. The signal distortion was caused by the acoustic properties of the skull layer. Considering the acoustic properties of the skull, it was found the object of the blood vessel became a pair of inverted half-circular shapes with the strong signal in the middle.

### 3.2. Analysis in Two Directions Under Specific Illumination of Pencil Beam

**Figure 5:**
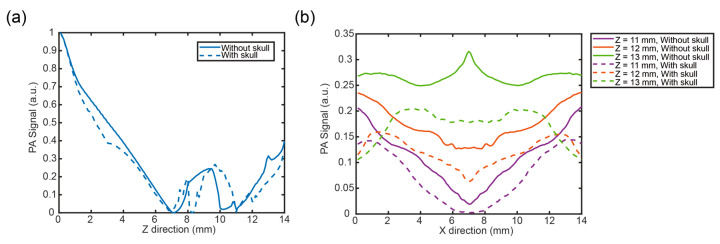
PA signals of reconstructed images without the skull or with the skull under the illumination of a pencil beam. (**a**) Z direction at *x* = 7 mm; (**b**) X direction at *z* = 11 mm, 12 mm, and 13 mm.

In this part, the PA signals of the reconstructed images under the specific illumination were compared with or without the skull. According to the results, it can be found that reconstructed images under the different illuminations had the same tendency. Thus, the results under the illumination of a pencil beam in Figure 5 were analyzed for an example.

Figure 5a shows the PA signals of the model without or with the skull. Firstly, let us see the results without the influence of the skull. The PA signals decreased in the layers of the scalp and the skull. Before leaving the skull layer, the PA signals arrived at the minimum value and then increased to the boundary, which may be caused by the artifacts and signal attenuation. In the CSF layer (at the depth of 8~10 mm), the tendency of the PA signals increased and then decreased. In the layer of the gray matter (at the depth of 10~12 mm), the PA signals became stronger with the depth increasing. Meanwhile, we can find it has small fluctuations at two boundaries of the blood vessel. For the results with the influence of the skull, there was an obvious change at the boundary of the scalp and skull layers (at the depth of 3 mm). Around the boundary of the skull and CSF layers (at the depth of 8 mm), the PA signal decreased to a very low value and then changed like the PA signal without the skull properties. The fluctuation may be due to the artifacts and signal attenuation caused by the interaction of the limited-view linear ultrasound sensor and the influence of the skull with other tissues.

According to Figure 5b, the change in the PA signals in the X direction can be analyzed. It can be known that the PA signals with or without the skull in the X direction had a large difference. Without the skull, the PA signals in the deeper region of the bottom boundary of the blood vessel (*z* = 13 mm) had the strongest intensity, and the second stronger PA signals were in the middle position of the blood vessel (*z* = 12 mm). The smallest PA signals were in the upper boundary of the blood vessel (*z* = 11 mm). From the results, we can know that without the influence of the skull, the PA signals showed stronger intensity in the deeper region, which is consistent with the results without the skull in Figure 5a. Meanwhile, we can find that the PA signals considering the skull had similar tendencies to the PA signals without the skull but with lower intensity, which may due to the loss of the signal propagation and reconstruction.

### 3.3. Photoacoustic Signal Distribution in Two Directions

To compare the influence of the illumination of different types of optical sources, the PA signals in the Z and X directions under the illumination of sixteen optical source types were figured in this part.

**Figure 6:**
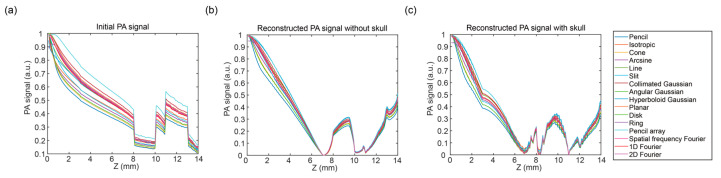
PA signals in the Z direction (*x* = 7 mm) under the illumination of sixteen optical source types. (**a**) Initial PA signal, (**b**) reconstructed PA signal without the skull, and (**c**) reconstructed PA signal with the skull.

Figure 6 shows the PA signals in the Z direction (*x* = 7 mm) under the illumination of sixteen optical source types. At the surface region within the scalp layer (<3 mm, as shown in Figure 6), there were differences in the PA signals under sixteen optical source types, which depend on the properties of the optical source types. In the deeper region (>3 mm), the PA signals had a consistent tendency under sixteen optical source illuminations due to the optical scattering and diffusion. In Figure 6a, the initial PA signals showed obvious layer characteristics, while the layer characteristics of the reconstructed PA signals without the skull in Figure 6b and with the skull in Figure 6c were not so obvious due to the acoustic attenuation. Meanwhile, after acoustic simulation and image reconstruction, the PA signals displayed obvious changes around the boundary of the layers, which may be caused by the stepped change in the initial PA signals.

**Figure 7:**
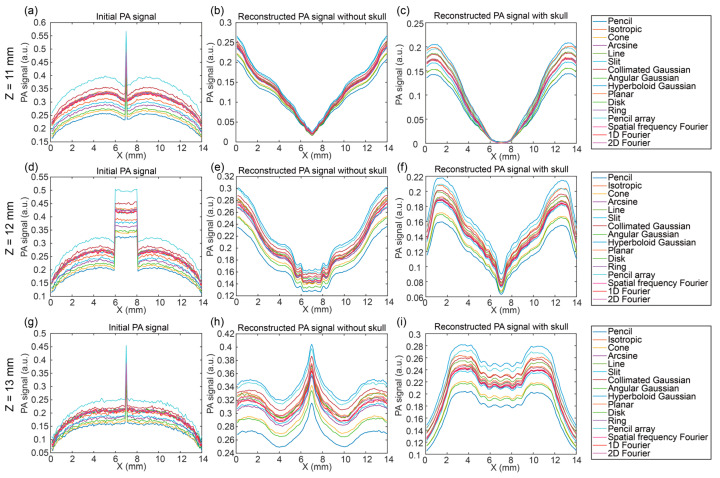
PA signals in the X direction at different Z positions under the illumination of sixteen optical source types. In the X direction at *z* = 11 mm (**a**–**c**): (**a**) initial PA signal, (**b**) reconstructed PA signal without the skull, and (**c**) reconstructed PA signal with the skull; in the X direction at *z* = 12 mm (**d**–**f**): (**d**) initial PA signal, (**e**) reconstructed PA signal without the skull, and (**f**) reconstructed PA signal with the skull; in the X direction at *z* = 13 mm (**g**–**i**): (**g**) initial PA signal, (**h**) reconstructed PA signal without the skull, and (**i**) reconstructed PA signal with the skull.

Figure 7 depicts the PA signals in the X direction at different Z positions (*z* = 11 mm, 12 mm, and 13 mm) under the illumination of sixteen optical source types. Similar to what was found in Figure 6, the PA signals in the deep region in the X direction had consistent changes with different intensities under different illuminations. The initial PA signal in the *z* = 11 mm and *z* = 13 mm showed obvious peaks, which indicates the strong signals of the blood vessel at the top and bottom boundaries. Meanwhile, the initial PA signals in the *z* = 12 mm were even in the region of the blood vessel. The reconstructed PA signals without the skull still had peaks in the *z* = 11 mm and 13 mm, and even in the *z* = 12 mm. The reconstructed PA signals with the skull in Figure 7i showed the same tendencies and characteristics as the reconstructed PA signal without the skull. Furthermore, the signal at the blood region in the reconstructed PA signal with the skull was lower than the surrounding area, while the signal at the blood region in the reconstructed PA signal without the skull was larger than the surrounding region. The left and right boundaries of the reconstructed PA signal with and without the skull showed different tendencies due to acoustic distortion and movement.

**Figure 8:**
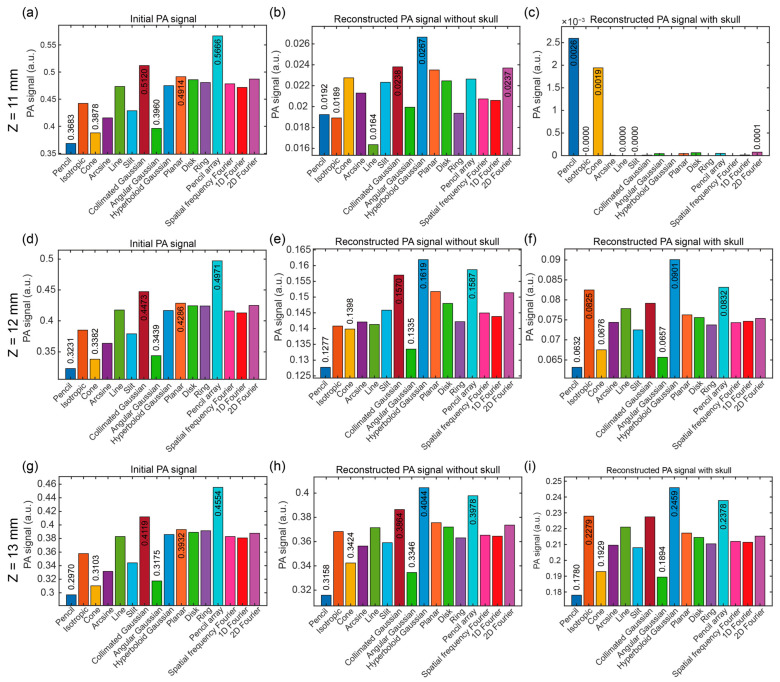
Maximum and minimum PA signals and corresponding optical source types at the specific position (*x* = 7 mm, *z* = 11, 12, and 13 mm) under the illumination of sixteen optical source types. In the X direction at *z* = 11 mm, (**a**–**c**): (**a**) initial PA signal, (**b**) reconstructed PA signal without the skull, and (**c**) reconstructed PA signal with the skull; in the X direction at *z* = 12 mm, (**d**–**f**): (**d**) initial PA signal, (**e**) reconstructed PA signal without the skull, and (**f**) reconstructed PA signal with the skull; in the X direction at *z* = 13 mm, (**g**–**i**): (**g**) initial PA signal, (**h**) reconstructed PA signal without the skull, and (**i**) reconstructed PA signal with the skull. The data of the first three maximum and the first three minimum PA signals were noted around the bars in the subfigures (**a**–**i**).

Figure 8 shows the maximum and minimum PA signals and corresponding optical source types at the specific position (*x* = 7 mm, *z* = 12 mm) under the illumination of sixteen optical source types. Based on Figure 8a,d,g, we found that the optical source types of the maximum and minimum PA signals were the same in the different Z positions (*z* = 11, 12, and 13 mm). However, the optical source types in the reconstructed PA signals varied in the different Z positions from the initial PA signals, which indicates that the PA changed during the propagation and receiving. At *z* = 11 mm, the optical source types were different in the reconstructed PA signal without and with the skull. The optical source types were the same but with different intensities in the reconstructed PA signal without and with the skull at *z* = 12 mm and 13 mm. Those results indicated the acoustic simulation makes the PA propagation more complex.

### 3.4. Discussion of Optical Source Types at Different Positions

In a summary of the results, the optical source types and related PA signals at the first maximum and minimum values for the initial PA signals, the reconstructed PA signals without the skull, and the reconstructed PA signals with the skull were summarized in Table 2, Table 3 and Table 4, respectively.

Table 2 shows the optical source types of the first three maximum and minimum of the initial PA signals. The optical source types of the first three maximum of the initial PA signals were all pencil array, collimated Gaussian, and planar sources. The optical source types of the first three minimum of the initial PA signals were all pencil, cone, and angular Gaussian sources. The first three maximum of the initial PA signals at the different positions had the same optical source types, as well as the first three minimum of the initial PA signals.

Table 3 shows the optical source types of the first three maximum and minimum of the reconstructed PA signals without the skull. Unlike the initial PA signals, we found that there were different optical source types of the first three maximum and minimum values in this model. The optical source type of the first maximum PA signals at different positions was all hyperboloid Gaussian sources. The optical source types with the second maximum PA signals were pencil array (twice), and collimated Gaussian source (once). The optical source types with the third maximum PA signals were the collimated Gaussian source (twice), and 2D Fourier source (once). In addition, the optical source type of the first minimum PA signals at different positions were pencil source (twice), and line source (once). The optical source types with the second minimum PA signals were angular Gaussian source (twice), and isotropic source (once). The optical source types with the third minimum PA signals were cone source (twice), and pencil source (once).

Table 4 shows the optical source types of the first three maximum and minimum of the reconstructed PA signals with the skull. It was found that the optical source types with the maximum PA signals at *z* = 11 mm were pencil, cone, and 2D Fourier sources and the optical source types with the minimum PA signals were isotropic, line, and slit sources, which are different from the model without the skull. However, the optical source types at *z* = 12 mm and 13 mm were the same as the model without the skull. Furthermore, the optical source types with the maximum PA signals were hyperboloid Gaussian, pencil array, and isotropic sources while the optical source types with the minimum PA signals were pencil, angular Gaussian, and cone sources. Therefore, the optical source types with the top three maximum PA signals were hyperboloid Gaussian source (twice), pencil array source (twice), and isotropic source (twice). In addition, the optical source types of the top three minimum PA signals at different positions were pencil source (twice), angular Gaussian source (twice), and cone source (twice).

In this research, 16 optical source types were simulated and compared while pencil beam [25], collimated Gaussian [26], and planar source [15] are usually used for simulation work to represent the real conditions. Although not all optical source types are often used in realistic conditions, the optical source can be designed and improved based on the specific requirements of the PAI system in realistic situations. For example, a pencil beam can be used for deep imaging with good focus while hyperboloid Gaussian can provide larger diffusion.

For brain imaging in the usage scenario of PAI, the skull would not only cause signal aberration and attenuation, but also cause signal reverberation, which would degrade the image quality. Meanwhile, the optical and acoustic simulation in this research used identical grid sizes (0.1 mm) which could oversimplify or misrepresent the physical phenomena. Optical simulations typically conduct the scattering model with sub-millimeter precision (0.1 mm or less), while acoustic simulations set grid sizes based on the requirement of the spatial resolutions related to the wavelength when dealing with wave propagation. Therefore, the more complex brain model can be researched in further work and the grid size can be analyzed to enhance the precision of the results.

Moreover, the linear ultrasound array combined with the DAS algorithm was used in the simulation of a 14 × 14 × 14 mm^3^ volume model. The limited-view linear arrays will cause the artifacts of the reconstructed images. Considering the whole brain model, the other arrays like circular or spherical arrays can be considered for PA signal acquisition to remove the artifacts caused by the ultrasound arrays.

## 4. Conclusions

The skull is a great challenge for PAI for human beings, with the high optical scattering and acoustic attenuation. To explore and improve photoacoustic generation and propagation, we conducted the photoacoustic simulation and image reconstruction of the multi-layer brain model with an embedded blood vessel under different optical source types. Based on the optical simulation results under different types of optical sources, this paper explored the characteristics of reconstructed images obtained from acoustic simulations with and without skull conditions. Specifically, we focused on the detection of blood vessels and evaluated the image reconstruction features, morphological characteristics, and intensity variations in the target vessels using optical and acoustic simulations. The results showed that under the initial PA signals, the types of optical source types corresponding to the strongest and weakest photoacoustic signals at different positions within the target region were consistent, while the optical source types were different in the reconstructed images. This study revealed the characteristics of acoustic signal transmission with and without skull conditions and its impact on image reconstruction. It further provides a theoretical basis for the selection of optical sources.

## Figures and Tables

**Table 1:** Optical properties of each brain tissue for simulation at the optical wavelength of 800 nm.

Brain Tissues	Absorption Coefficient, μ_a_ (1/mm)	Scattering Coefficient, μ_s_ (1/mm)	Anisotropy Factor, g	Refractive Index, n
Scalp	0.018	19.0	0.9	1.37
Skull	0.016	16.0	0.9	1.43
Cerebrospinal fluid	0.004	2.4	0.9	1.33
Gray matter	0.036	22.0	0.9	1.37
Blood vessel	0.223	50.0	0.99	1.4

**Table 2:** Optical source types of the first three maximum and minimum of the initial PA signals.

Position	Optical Sources of the First Three Maximum PA Signals	Optical Sources of the First Three Minimum PA Signals
*z* = 11 mm, *x* = 7 mm	1. Pencil array (0.5666 a.u.) 2. Collimated Gaussian (0.5120 a.u.) 3. Planar (0.4914 a.u.)	1. Pencil (0.3683 a.u.) 2. Cone (0.3878 a.u.) 3. Angular Gaussian (0.3960 a.u.)
*z* = 12 mm, *x* = 7 mm	1. Pencil array (0.4971 a.u.) 2. Collimated Gaussian (0.4473 a.u.) 3. Planar (0.4286 a.u.)	1. Pencil (0.3231 a.u.) 2. Cone (0.3382 a.u.) 3. Angular Gaussian (0.3439 a.u.)
*z* = 13 mm, *x* = 7 mm	1. Pencil array (0.4554 a.u.) 2. Collimated Gaussian (0.4119 a.u.) 3. Planar (0.3932 a.u.)	1. Pencil (0.2970 a.u.) 2. Cone (0.3103 a.u.) 3. Angular Gaussian (0.3175 a.u.)

**Table 3:** Optical source types of the first three maximum and minimum of the reconstructed PA signals without the skull.

Position	Optical Sources of the First Three Maximum PA Signals	Optical Sources of the First Three Minimum PA Signals
*z* = 11 mm, *x* = 7 mm	1. Hyperboloid Gaussian (0.0267 a.u.) 2. Collimated Gaussian (0.0238 a.u.) 3.2D Fourier (0.0237 a.u.)	1. Line (0.0164 a.u.) 2. Isotropic (0.0189 a.u.) 3. Pencil (0.0192 a.u.)
*z* = 12 mm, *x* = 7 mm	1. Hyperboloid Gaussian (0.1619 a.u.) 2. Pencil array (0.1587 a.u.) 3. Collimated Gaussian (0.1570 a.u.)	1. Pencil (0.1277 a.u.) 2. Angular Gaussian (0.1335 a.u.) 3. Cone (0.1398 a.u.)
*z* = 13 mm, *x* = 7 mm	1. Hyperboloid Gaussian (0.4044 a.u.) 2. Pencil array (0.3978 a.u.) 3. Collimated Gaussian (0.3864 a.u.)	1. Pencil (0.3158 a.u.) 2. Angular Gaussian (0.3346 a.u.) 3. Cone (0.3424 a.u.)

**Table 4:** Optical source types of the first three maximum and minimum of the reconstructed PA signals with the skull.

Position	Optical Sources of the First Three Maximum PA Signals	Optical Sources of the First Three Minimum PA Signals
*z* = 11 mm, *x* = 7 mm	1. Pencil (0.0026 a.u.) 2. Cone (0.00019 a.u.) 3.2D Fourier (0.0001 a.u.)	1. Isotropic (0.0000 a.u.) 2. Line (0.0000 a.u.) 3. Slit (0.0000 a.u.)
*z* = 12 mm, *x* = 7 mm	1. Hyperboloid Gaussian (0.0901 a.u.) 2. Pencil array (0.0832 a.u.) 3. Isotropic (0.0825 a.u.)	1. Pencil (0.0632 a.u.) 2. Angular Gaussian (0.0657 a.u.) 3. Cone (0.0676 a.u.)
*z* = 13 mm, *x* = 7 mm	1. Hyperboloid Gaussian (0.2459 a.u.) 2. Pencil array (0.2378 a.u.) 3. Isotropic (0.2279 a.u.)	1. Pencil (0.1780 a.u.) 2. Angular Gaussian (0.1894 a.u.) 3. Cone (0.1929 a.u.)

## Data Availability

The datasets generated for this study are available on request to the corresponding authors.
